# Wavelength of a Turing-type mechanism regulates the morphogenesis of meshwork patterns

**DOI:** 10.1038/s41598-021-84313-7

**Published:** 2021-03-01

**Authors:** Shan Guo, Ming-zhu Sun, Xin Zhao

**Affiliations:** 1 Institute of Robotics and Automatic Information Systems, Nankai University, College of Artificial Intelligence, 201-02, Tianjin, 300350 People’s Republic of China; 2 Tianjin Key Laboratory of Intelligent Robotics, Nankai University, Tianjin, 300350 People’s Republic of China

**Keywords:** Morphogenesis, Pattern formation, Biochemical reaction networks

## Abstract

The meshwork pattern is a significant pattern in the development of biological tissues and organs. It is necessary to explore the mathematical mechanism of meshwork pattern formation. In this paper, we found that the meshwork pattern is formed by four kinds of stalk behaviours: stalk extension, tip bifurcation, side branching and tip fusion. The Turing-type pattern underlying the meshwork pattern is a Turing spot pattern, which indicates that the Turing instability of the spot pattern promotes activator peak formation and then guides the formation of meshwork patterns. Then, we found that the Turing wavelength decreased in turn from tip bifurcation to side branching to tip fusion via statistical evaluation. Through the functional relationship between the Turing wavelength and model parameters ($\upvarepsilon ,{ \rho }_{A}$ and ${\rho }_{H}$), we found that parameters $\upvarepsilon$ and ${\rho }_{H}$ had monotonic effects on the Turing wavelength and that parameter ${\rho }_{A}$ had nonmonotonic effects. Furthermore, we performed simulations of local meshwork pattern formation under variable model parameter values. The simulation results verified the corresponding relationship between the Turing wavelength and stalk behaviours and the functional relationship between the Turing wavelength and model parameters. The simulation results showed that the Turing wavelength regulated the meshwork pattern and that the small Turing wavelength facilitated dense meshwork pattern formation. Our work provides novel insight into and understanding of the formation of meshwork patterns. We believe that studies associated with network morphogenesis can benefit from our work.

## Introduction

Meshwork patterns are a significant pattern for the development of biological tissues and organs, such as alveolar microvascular networks, which are very important for nutrient transport and gas exchange. Our previous work simulated the generation of meshwork patterns based on an activator-inhibitor reaction–diffusion model^1^. However, the formation of a meshwork pattern is a complicated process that is formed by sprouting, sprout extension and anastomosis^2^. It is not easy to explain the transformation of stalk behaviour in the process of meshwork pattern formation by the interaction between morphogens. To further explain meshwork pattern formation, the mathematical mechanism underlying the meshwork pattern is explored in this paper.

A mathematical model—the Meinhardt model^3^—is used in this paper to investigate the mechanism of meshwork pattern formation. The Meinhardt model is a reaction–diffusion model based on molecular mechanisms. It describes the interaction between morphogens. Some other models of meshwork morphogenesis based on other mechanisms also exist. For example, the Murray model is based on a mechanical mechanism that describes meshwork pattern formation through cell aggregation in the culture system^4^, and the Chaplain-Anderson model is based on a cellular mechanism that describes meshwork pattern formation by multigenerational tip bifurcation under exogenous stimulation^2^. Compared with the Murray model and Chaplain-Anderson model, the Meinhardt model can perform similar simulation results^5,6^, perform more modes of branching (side branching and tip bifurcation)^7^, and form complex patterns by branch growth. Based on the model, the meshwork pattern was performed in our previous work. In a spherical shell domain, a tree-like pattern is first formed by multigenerational branches and then transferred to the meshwork pattern through branch tip fusion^1^. In this paper, we aim to further explore the mathematical mechanism underlying meshwork pattern formation.

The Meinhardt model used in this paper is a reaction–diffusion model based on the Turing activator-inhibitor theory^3^. The Turing-type model uses dynamic interactions between activator and inhibitor molecules to determine the wavelength of patterns and produces periodic patterns of spots or stripes by Turing instability^8–13^. In nature, the formation of repetitive structures is often consistent with a reaction–diffusion mechanism, or the Turing model, of self-organizing systems^14,15^. For example, Hox genes regulate the polydactyly in mice by controlling the wavelength of a Turing-type mechanism^16^. Therefore, Turing mechanism research is an effective way to explore the formation of patterns. In our previous study on branching patterns, Turing instability was shown in the dynamics of side branching and tip bifurcation^6^, and the Turing wavelength underlying the branching patterns was explored^17^. In this paper, we conduct Turing instability analysis to reveal the mathematical mechanism underlying the meshwork pattern.

The formation of meshwork patterns is a complicated process. Various stalk behaviours occur in the process of meshwork pattern formation, such as sprouting, sprout growth, sprout splitting and anastomosis. In fact, these stalk behaviours exist widely in the network structure development of biological tissues and organs, such as the sprout growth of a mosaic embryoid body in vitro (Supplementary Fig. S1a)^18,19^, arterial arborescence in the intracortical capillary networks from the collateral sulcus in the temporal lobe (Supplementary Fig. S1b)^20^, intersegmental vessel (ISV) sprouting from the dorsal aorta in zebrafish embryos (Supplementary Fig. S1c)^18,21^, and vessel fusion of the dorsal longitudinal anastomotic vessel (DLAV) in zebrafish embryos (Supplementary Fig. S1d)^21^. As the meshwork structure is composed of these stalk behaviours, we attempt to discern the Turing characteristics of meshwork patterns by exploring the Turing characteristics of stalk behaviours.

In this paper, we aimed to explore the mathematical mechanism of meshwork patterns through Turing instability analysis. First, we performed local meshwork pattern formation, including four kinds of stalk behaviours, and explored the Turing-type patterns underlying the stalk behaviours. Second, we explored the Turing wavelength corresponding to the stalk behaviours through statistical evaluation. Third, we obtained the method of adjusting the Turing wavelength by the functional relationships between the Turing wavelength and model parameters. Then, we explored the influences of model parameters on meshwork pattern formation, which verified the regulatory function of the Turing wavelength on meshwork patterns.

## Methods

### Mathematical model

Our mathematical model consists of a set of partial differential equations for reaction and diffusion between biochemical morphogens, as shown in Eqs. (1–4)^3^. There were four variables in the model: activator *A*, inhibitor *H*, substrate *S*, and cell differentiation marker *Y*. Variable *Y* is the marker for recording the state of cell differentiation (Y ~ 0 means undifferentiated state, Y ~ 1 means differentiated state).

1 $$ \frac{\partial A}{{\partial t}} = \frac{{cA^{2} S}}{H} - \mu A + \rho_{A} Y + D_{A} \nabla^{2} A, $$

2 $$ \frac{\partial H}{{\partial t}} = cA^{2} S - vH + \rho_{H} Y + D_{H} \nabla^{2} H, $$

3 $$ \frac{\partial S}{{\partial t}} = c_{0} - \gamma S - \varepsilon YS + D_{S} \nabla^{2} S, $$

4 $$ \frac{\partial Y}{{\partial t}} = dA - eY + \frac{{Y^{2} }}{{1 + fY^{2} }}. $$

This model assumes that activator and inhibitor mutually react and diffuse under the participation of substrate, form local high concentration signals of activator, and then induce cell differentiation to form the structure. In the model, Eq. (1) indicates that activator *A* is produced in autocatalytic reaction kinetics with a dependence on substrate *S* and is simultaneously inhibited by inhibitor *H* ($\frac{{cA^{2} S}}{H}$), and activator *A* is concurrently secreted by differentiated cells *Y* at rate $\rho_{A}$ ($\rho_{A} Y$), diffuses with diffusion coefficient $D_{A}$ ($D_{A} \nabla^{2} A$)$\mathrm{A}$, and degrades at rate $\mu$ ($- \mu A$); Eq. (2) indicates that inhibitor *H* is catalysed by activator *A* with a dependence on substrate *S* ($cA^{2} S$), secreted by differentiated cells *Y* at rate $\rho_{H}$ ($\rho_{H} Y$), diffuses with diffusion coefficient $D_{H}$ ($D_{H} \nabla^{2} H$), and degrades at rate $v$ ($- \nu H$); Eq. (3) indicates that substrate *S* is produced at a constant rate ${c}_{0}$, is consumed by differentiated cells *Y* at rate $\varepsilon$ ($- \varepsilon YS$), diffuses with diffusion coefficient $D_{S}$ ($D_{S} \nabla^{2} S$), and degrades at rate $\gamma$ ($- \gamma S$); Eq. 4 indicates that marker *Y* of cell differentiation is activated by high concentrations of activator *A* ($dA$), degrades at rate *e* ($- eY$), and has a positive feedback effect on itself, and this effect will be saturated at high concentration of *Y* ($\frac{{Y^{2} }}{{1 + fY^{2} }}$).

### Turing instability underlying the model

In our mathematical model, the interaction between cell differentiation marker Y and substrate S produced the spatial extension of the Y-stalk, and the interaction between activator A and inhibitor H formed the local pattern on the stalk. Therefore, the model could be decoupled into two semi-independent subsystems: classical activator-inhibitor dynamics (A/H local dynamics) and extension of the Y-stalk (Y/S dynamics). The Turing instability was exhibited by the local pattern formed by the A/H local dynamics. Thus, we used the decoupled model of A/H local dynamics to explore the Turing instability underlying the meshwork patterns.

**Figure 1:**
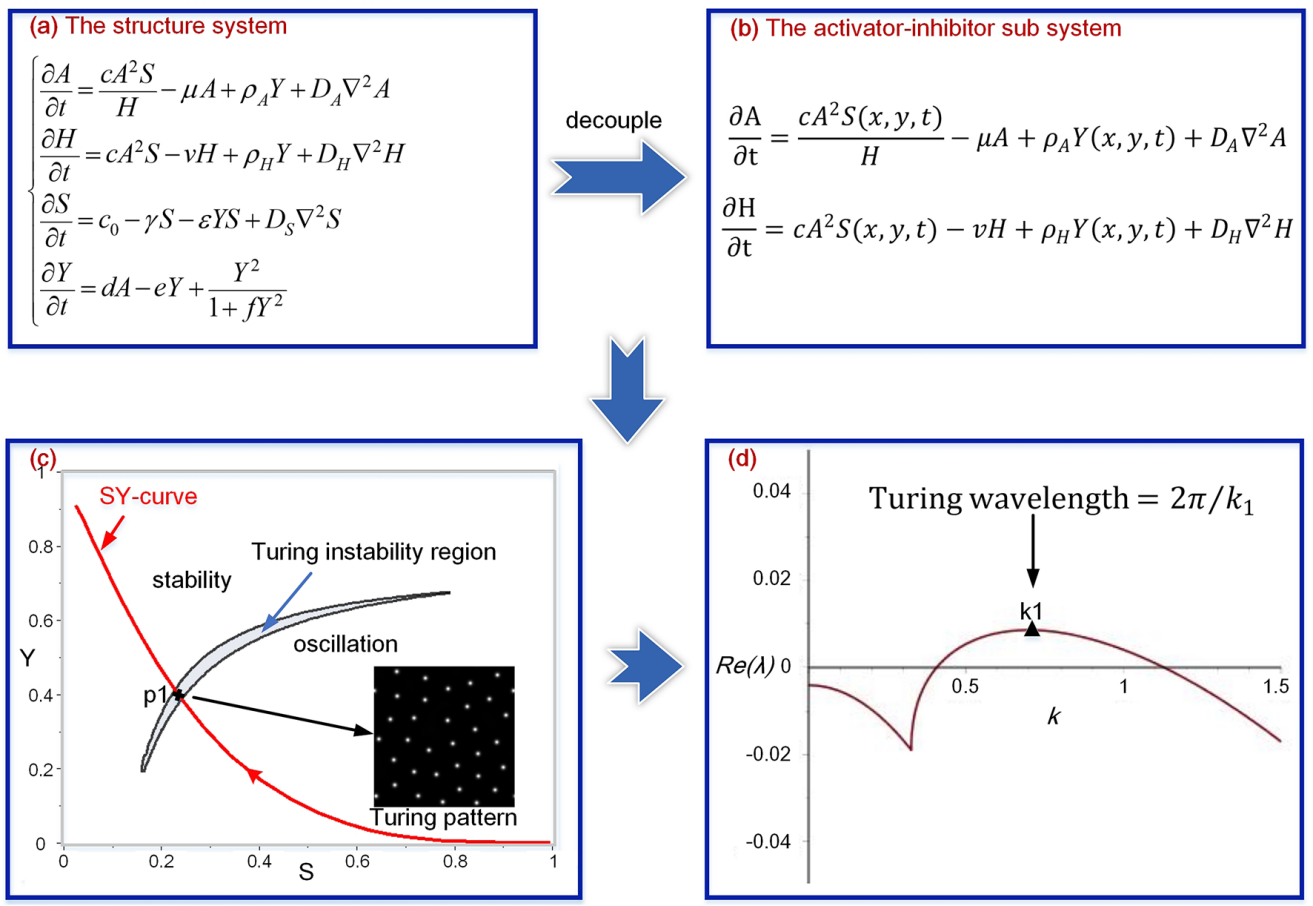
Schematic diagram of the Turing instability research scheme. (**a**) The mathematical model for meshwork pattern formation. (**b**) The decoupled model of the activator-inhibitor subsystem. (**c**) A crescent-shaped Turing region is presented in the S–Y parameter space of the activator-inhibitor model. The red curve is the SY curve for the cell differentiation trajectory at the site in the meshwork structure. Point p1 indicates the cell differentiation state with Turing instability, the (S, Y) pair of which is substituted into the activator-inhibitor subsystem to acquire the Turing-type pattern. In the Turing pattern, black indicates a low concentration of activator, while white indicates a high concentration of activator. (**d**) The dispersion relation for the Turing pattern. *k*1 is the critical wavenumber at which the maximum value of $\mathrm{Re}\left(\lambda \right)$ occurs. The Turing wavelength is calculated by dividing 2$\uppi$ by the critical wavenumber *k*1.

The model of the activator-inhibitor subsystem was decoupled from the 4-variable model by making *Y* and *S* controllable parameters in the local A/H dynamics (as depicted in Fig. 1a,b). When the Turing instability region (see Supplement Information for the Turing instability analysis) and the differentiation trajectory of a cell (SY-curve) were achieved, a (S, Y) pair had to be obtained at the centre of the overlapping segment of the SY-curve and Turing instability region. Then, the (S, Y) pair was substituted into parameters *S* and *Y* of the A/H subsystem, and the Turing-type pattern could be obtained by the A/H subsystem model (as depicted in Fig. 1c). A Turing pattern corresponds to a Turing wavelength. The dispersion relations describe a function of $\mathrm{Re}\left(\lambda \right)$ on wavenumber $k$, where $\lambda$ is the eigenvalue with the largest real part (see Supplement Information for obtaining the dispersion relations). The Turing wavelength was calculated by dividing $2\uppi$ by the critical wavenumber at which the maximum value of $\mathrm{Re}\left(\lambda \right)$ occurred (as depicted in Fig. 1d).

### Numerical simulation

#### Simulation environment settings of meshwork patterns

In this paper, the simulation environment settings of meshwork patterns were basically the same as those in our previous works^1,7^. A two-dimensional (2D) square domain was established for local meshwork pattern formation. The square computing space was orthogonally discretized into a uniform grid with a space step $\Delta x=\Delta y=0.3$. The grid size was 100 × 100. The finite-difference scheme was used for spatial discretization. The time step was $\Delta x\times \Delta y\times 0.4\times {D}_{H}$. The initial conditions of the simulations were set as follows. In the growth domain, activator, inhibitor and substrate were uniformly distributed. The concentrations of activator and inhibitor were set to very small values: $A = 0.001$, $H = 0.01$, the concentrate of substrate was set to a high value: $S = 1.0$. The initial growth site of structures was represented by $Y = 1$ in a small region, and the other part of the domain was set as $Y = 0$. Then, the model was numerically simulated using a forward Euler method with no-flux boundary conditions. The diffusion operator was a four-point Laplacian on a uniform Cartesian grid. The stalk growth was described by converting variable *Y* from $Y = 0$ to $Y = 1$ in the presence of high concentrations of activator (the $dA$ term in Eq. 4)*.*

### Simulation environment settings of Turing patterns

The Turing pattern was performed in a 2D square domain based on the model of the activator-inhibitor subsystem (Eqs. 1 and 2). The model was numerically simulated using a forward Euler method with periodic boundary conditions. The grid size was 200 $\times$ 200. The parameter values were set according to the full system. The initial values of the activator and inhibitor were set to $A=1.0$ and $H=0.01$. The simulation started from a randomly perturbed uniform initial condition and stopped when the stationary spatial pattern was formed.

## Results

### Turing spot patterns underlying the meshwork pattern

**Figure 2:**
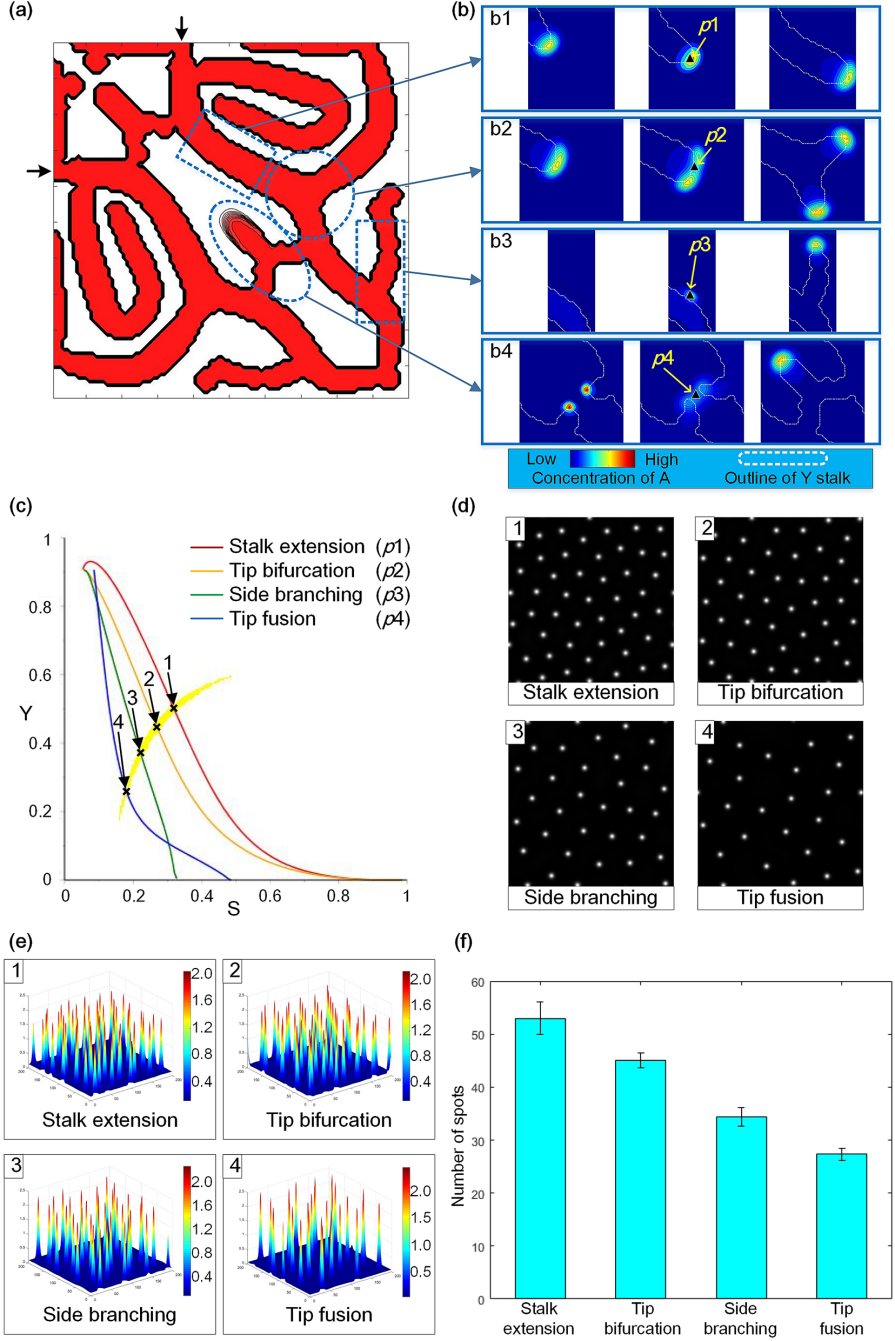
Turing patterns underlying the stalk behaviours for meshwork pattern formation. (**a**) The meshwork pattern formed in the square domain, which includes the four kinds of stalk behaviours: stalk extension, tip bifurcation, side branching and tip fusion. There are two initial growth sites located on the adjacent boundaries of the domain (marked by black arrows). In the image, red represents the generated meshwork structure, white represents the remaining region without structure generation, and black lines represent the outline of the structure. The blue dotted boxes indicate the areas where stalk behaviours occur. (**b**) The dynamic process of activator *A* during formation of the four stalk behaviours. (b1) stalk extension; (b2) tip bifurcation; (b3) side branching; (b4) tip fusion. Sites *p*1, *p*2, *p*3 and *p*4, marked by black triangles, indicate the typical sites of stalk behaviour. The differentiation process at these sites was used for further exploration of Turing characteristics. Blue represents a low concentration of activator *A*, red represents a high concentration of activator *A*, and the white dashed line indicates the outline of stalks. (**c**) The crescent-shaped Turing region and the SY curves of differentiation trajectories at the sites (*p*1, *p*2, *p*3 and *p*4 in (**b**)) in the four stalk behaviours. Points (1, 2, 3 and 4, marked by ‘**×**’) indicate the cell differentiation state with Turing instability. The (S, Y) pairs of these points are used to acquire the Turing-type pattern. (**d**) The underlying Turing patterns of the four stalk behaviours. The Turing patterns are obtained by the A/H submodel when the (S, Y) pairs of points (1, 2, 3 and 4 in (**c**)) are substituted into the model. Black represents a low concentration of activator A, and white represents a high concentration of activator *A*. (**e**) The Turing patterns underlying the four stalk behaviours in 3D form. (**f**) The number of spots in the underlying Turing spot patterns of stalk behaviours. The mean and standard deviation of each stalk behaviour are calculated from ten Turing patterns with random initial disturbance. (Parameters: ${D}_{A}=0.02, { D}_{H}=0.26, { D}_{s}=0.06,\mathrm{ c}=0.002,{ c}_{0}=0.02,\upmu =0.16,\mathrm{ v}=0.04,\upgamma =0.02,\upvarepsilon =0.475,\mathrm{ d}=0.008,\mathrm{ e}=0.1,\mathrm{ f}=10, { \rho }_{A}=0.03, {\rho }_{H}=0.00005$).

Meshwork pattern formation is a complicated process. The network structures in biological tissues and organs include various stalk behaviours, such as sprouting, sprout growth, sprout splitting and anastomosis, as shown in Supplementary Fig. S1^18–21^. In this section, a local meshwork pattern formation (Fig. 2a) was performed in a 2D square domain. There were four kinds of typical stalk behaviours in the meshwork pattern: stalk extension, tip bifurcation, side branching, and branch tip fusion (Fig. 2b). These stalk behaviours corresponded to sprouting, sprout growth, sprout splitting and anastomosis in biological network structures. The simulation result of the meshwork pattern confirmed that the meshwork pattern was formed by the four stalk behaviours. Then, the Turing patterns underlying the stalk behaviours for meshwork pattern formation were obtained.

The way to obtain Turing patterns is shown in Fig. 1c By calculating the S–Y parameter space of the A/H submodel for Turing instability, the Turing region was obtained, as shown in Fig. 2c. The SY curves (shown in Fig. 2c recording cell differentiation trajectories) of typical sites representing stalk behaviours were also plotted in the S–Y plane. Then, the simulation results of the Turing patterns (Fig. 2d) underlying the stalk behaviours were obtained by the A/H submodel, and the 3-dimensional (3D) form of the Turing patterns is shown in Fig. 2e. The density histogram of Turing patterns is shown in Fig. 2f.

Figure 2a shows the structure of a local meshwork pattern in a 2D square domain. This structure is generated from the two initial growth sites, which are located on the adjacent sides of the square domain. In the structure, there are four kinds of stalk behaviours for meshwork pattern formation: stalk extension, tip bifurcation, side branching and tip fusion. Figure 2b shows the dynamic process of the formation of the four stalk behaviours. The typical sites (*p*1–*p*4) of each stalk behaviour are marked by black triangles, such as the site on the stalk extension trajectory, the bifurcation site where one stalk splits into two, the sprouting site where a new lateral branch occurs, and the fusion site where two stalks merge into one. These sites represent the four kinds of stalk behaviours. Then, the SY curves of the cell differentiation trajectories of the stalk behaviours are extracted from the typical sites (as shown in Fig. 2c). Moreover, the crescent-shaped Turing region of the model is also plotted in the S–Y plane in Fig. 2c. The points (1,2,3,4 marked by ‘**×**’) are located at the centre of the overlapping segments of SY-curves and the Turing region. By extracting the (S, Y) pairs of these points and substituting them into the A/H subsystem, the Turing-type pattern can be obtained by simulation of the A/H submodel. Figure 2d shows the Turing patterns underlying the four stalk behaviours, which are all spot patterns. Figure 2e shows the Turing spot patterns in 3D form. The spot patterns are in the form of peaks of activator concentration. This result indicates that the Turing instability of stalk behaviours promotes the formation of activator peaks. As a high concentration of activator activates irreversible differentiation, structure formation is facilitated by Turing instability of the spot pattern. Figure 2f shows the number of spots of the underlying Turing pattern of stalk behaviours. The number of spots of Turing spot patterns is quite different between different stalk behaviours.

Above all, we conclude that the meshwork pattern is formed via four kinds of stalk behaviours: stalk extension, tip bifurcation, side branching and tip fusion; the Turing patterns corresponding to the stalk behaviours are all spot patterns, and thus, the Turing-type pattern underlying the meshwork pattern is a Turing spot pattern; the Turing instability of spot pattern promotes activator peak formation and then guides the formation of meshwork patterns; the density of Turing spot pattern is quite different among the four stalk behaviours.

### Turing wavelength underlying stalk behaviours of meshwork pattern formation

The meshwork pattern formation includes various stalk behaviours: stalk extension, tip bifurcation, side branching and tip fusion. These stalk behaviours have the same Turing-type pattern: Turing spot pattern (as shown in Fig. 2d,e). However, the spot density of the Turing spot pattern is quite different between the four stalk behaviours (as shown in Fig. 2f). This means that there are differences in the Turing characteristics of the four stalk behaviours. To quantitatively evaluate the Turing characteristics underlying the stalk behaviours, the Turing wavelength of each stalk behaviour is explored in this section because the Turing pattern is characterized by a critical wavelength^22^. The Turing wavelength was explored by dispersion relation analysis. The dispersion relations describe a function of Re(λ) that depends on wavenumber k, where λ is the eigenvalue with the largest real part (see Supplementary Information for obtaining the dispersion relations). The wavelength is calculated by dividing 2π by the critical wavenumber at which the maximum value of Re(λ) occurs.

**Figure 3:**
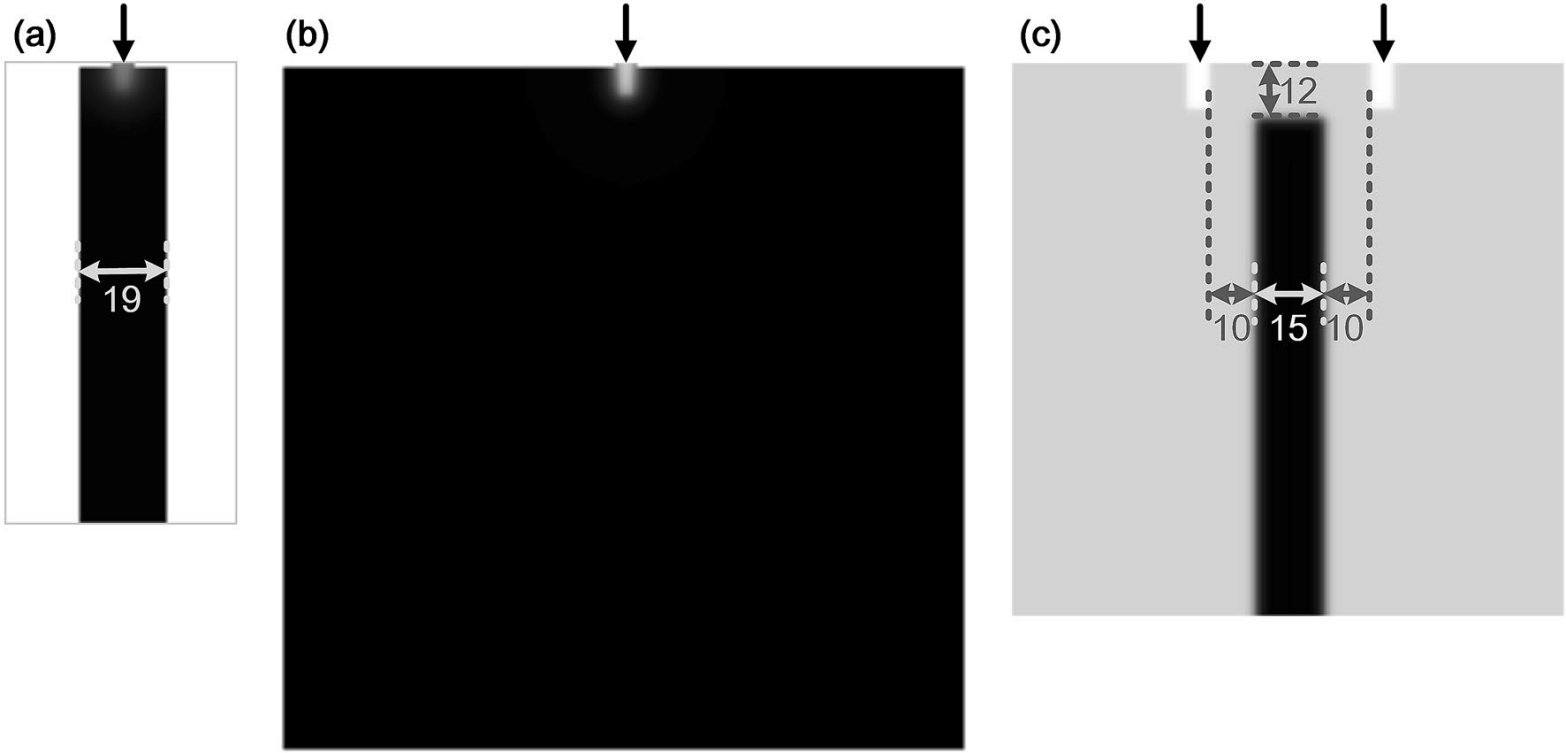
Simulation environment settings for each stalk behaviour formation. (**a**) Simulation environment for stalk extension. The black channel is the growth region for one stalk extension. The width of the channel is 19, and the grid size of the whole domain is $$50\times 100$$. In the black region, the substrate production rate is $${c}_{0}=0.02$$, and the initial value of the substrate is $${S}_{init}=1.0$$. (**b**) Simulation environment for tip bifurcation and side branching. The square region is set as the growth region, and the grid size is $$150\times 150$$. This domain is used for tip bifurcation when parameter ε is high and for side branching when ε is low. (**c**) Simulation environment for tip fusion. A local high concentration substrate region, set between the two initial growth sites, will drive stalk tips to meet and fuse. The grid size of the whole domain is $$120\times 120$$. In the local high concentration region (black strip), the substrate production rate is higher ($${c}_{0}=0.05)$$ and the initial value of substrate is $${S}_{init}={c}_{0}/\gamma $$ ($${S}_{init}=2.5$$), while in the other area, $${c}_{0}=0.02$$ and $${S}_{init}=1.0$$. The width of the strip region is 15. The distance between the strip region and the upper boundary of the growth domain is 12. The distance between the strip region and the initial growth sites in the horizontal direction is 10. The initial growth sites, set as small rectangular regions in which Y = 1, are indicated by the black arrows in (**a–c**).

To explore the Turing wavelength of stalk behaviours more conveniently, new simulation conditions were set for each stalk behaviour, as shown in Fig. 3. For stalk extension, an elongated rectangular channel was set as the growth domain for one stalk extension (black rectangular strip in Fig. 3a). The width of the channel was 19, and the grid size of the whole domain was $50\times 100$. Figure 3b shows the square space, which was set as the growth domain for tip bifurcation and side branching. The grid size of the domain was $150\times 150$. As the branching modes (tip bifurcation and side branching) are regulated by the parameter ε^7,17^, the square domain was used for tip bifurcation when ε was high and for side branching when ε was low. Figure 3c shows the simulation environment settings for branch tip fusion. Two initial growth sites were set on the top of a square domain, and a strip area with a higher concentration of substrate was set between the two initial growth sites to drive the branch tips to meet and fuse together. The grid size of the square domain was $120\times 120$. The width of the strip with a higher concentration of substrate was 15. The substrate production rate was set to ${c}_{0}=0.05$ in the strip area and ${c}_{0}=0.02$ in the other region.

**Figure 4:**
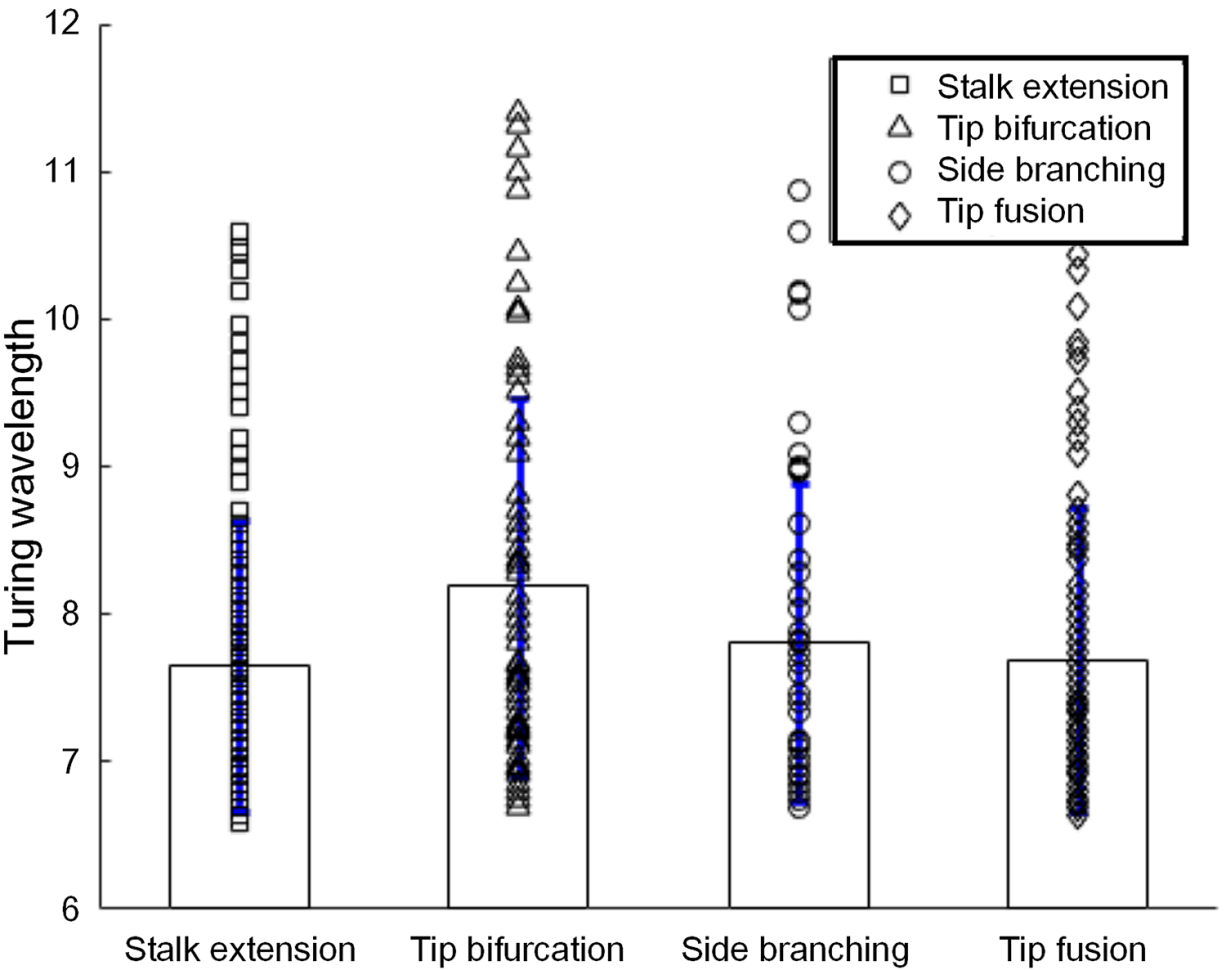
Turing wavelength of the four stalk behaviours under variable model parameter values. The scatter markers indicate the Turing wavelengths of each simulation of stalk behaviours. The histogram shows the mean and standard deviation of the Turing wavelength of each stalk behaviour. Parameter ρ_A_ ranges from 0.02 to 0.05 at interval 0.01, ρ_H_ ranges from 0.00005 to 0.00009 at interval 0.00001, and ε ranges from 0.10 to 0.30 at interval 0.05 for the mode of side branching and ranges from 0.5 to 0.9 at interval 0.1 for the mode of tip bifurcation.

Then, under the simulation conditions shown in Fig. 3, simulations of each stalk behaviour were performed with variable parameters (ε, ρ_A_ and ρ_H_). A large number of simulations of the four stalk behaviours were performed to explore the Turing wavelength corresponding to each stalk behaviour through statistical evaluation. Part of the simulation results are shown in Supplementary Figs. S2–S5. Through dispersion relation analysis, the Turing wavelengths for each simulation of stalk behaviours were obtained and are shown in Fig. 4. Table 1 records the sample size of each stalk behaviour and the mean and standard deviation of the Turing wavelength underlying stalk behaviours. Figure 4 shows the Turing wavelengths of the four stalk behaviours through statistical evaluation. The histogram intuitively indicates that the Turing wavelengths corresponding to the four stalk behaviours were different. Table 1 quantitatively shows the different Turing wavelengths underlying the stalk behaviours. Among the four stalk behaviours, tip bifurcation, side branching and tip fusion were closely related to the complexity of meshwork patterns. The Turing wavelength corresponding to tip bifurcation was the largest, and the Turing wavelength decreased in turn from tip bifurcation to side branching to tip fusion. The Turing wavelength of stalk extension was close to that of tip fusion. Therefore, we concluded that a larger Turing wavelength was more conducive to tip bifurcation, and a smaller Turing wavelength was more conducive to tip fusion.

**Table 1:** Turing wavelength of four kinds of stalk behaviours.

Type	Sample size	Mean of Turing wavelength	Standard deviation of Turing wavelength
Stalk extension	142	7.6493	0.9921
Tip bifurcation	84	8.1889	1.2713
Side branching	69	7.8114	1.0731
Tip fusion	124	7.6935	1.0351

### Relationship between Turing wavelength and model parameters

Figure 4 shows that the different stalk behaviours were facilitated by different Turing wavelengths. Stalk behaviours appeared to be regulated by the Turing wavelength and then the meshwork pattern. However, a way to control the Turing wavelength is needed before exploring the effects of the Turing wavelength on meshwork patterns. As the meshwork pattern is formed based on a mathematical model, a strategy for Turing wavelength regulation is to vary the values of the model parameters.

In our model, there were 14 different parameters, most of which were the biological parameters of morphogens, such as the diffusion coefficients (${D}_{A}, {D}_{H}, {D}_{s}$), degradation rates ($\upmu ,\mathrm{ v},\upgamma$) and autocatalytic rate of activator (c); these are certain for given morphogens^23^. For example, the effective diffusion coefficient of Dpp (a BMP homologue as activator) in tissue is (0.1 ± 0.05) × 10^−8^ cm^2^ s^–1^, and the Dpp degradation rate is (2.52 × 10^−4^) ± (1.29 × 10^−4^)s^−1^^23^. The parameters ($\mathrm{d},\mathrm{ e},\mathrm{ f}$) are used to describe the cell differentiation, which are set to certain values ($\mathrm{d}=0.008,\mathrm{ e}=0.1,\mathrm{ f}=10$). Therefore, the pattern is usually regulated by the remaining parameters (${c}_{0},\upvarepsilon ,{ \rho }_{A}, {\rho }_{H}$). These parameters (${c}_{0},\upvarepsilon ,{ \rho }_{A}, {\rho }_{H}$) have shown sensitive influences on patterns in our previous work^7^. Among them, parameter ${c}_{0}$ is the constant rate of substrate production, which provides the environment for stalk growth, while the other three parameters ($\upvarepsilon ,{ \rho }_{A}, {\rho }_{H}$) are directly related to stalks (parameter $\upvarepsilon$ is the substrate consumption rate by differentiated cells, parameter ${\rho }_{A}$ is the activator secretion rate by differentiated cells, and parameter ${\rho }_{H}$ is the inhibitor secretion rate by differentiated cells). Thus, these three parameters ($\upvarepsilon ,{ \rho }_{A}, {\rho }_{H}$) were selected to regulate the Turing wavelength in this paper. The relationships between the Turing wavelength and the three model parameters are explored in this section.

**Figure 5:**
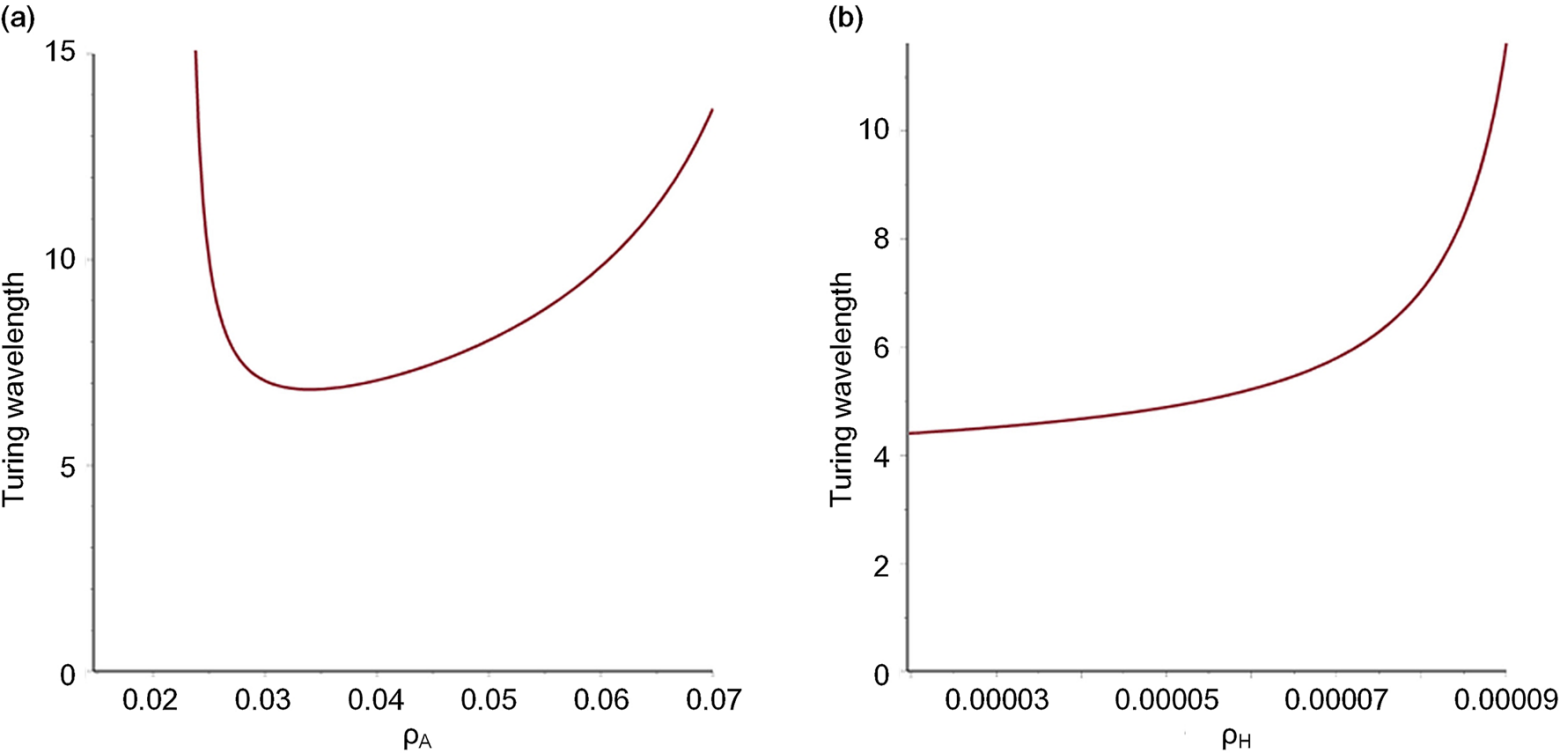
Function curves of the Turing wavelength on model parameters ${\rho }_{A}$ and ${\rho }_{H}$. (**a**) Wavelength/${\rho }_{A}$; (Other parameters: ${D}_{A}=0.02, { D}_{H}=0.26, { D}_{s}=0.06,\mathrm{ c}=0.002,{ c}_{0}=0.02,\upmu =0.16,\mathrm{ v}=0.04,\upgamma =0.02,\upvarepsilon =0.7,\mathrm{ d}=0.008,\mathrm{ e}=0.1,\mathrm{ f}=10, {\rho }_{H}=0.00008$.) (**b**) Wavelength/${\rho }_{H}$. (Other parameters: ${D}_{A}=0.02, { D}_{H}=0.26, { D}_{s}=0.06,\mathrm{ c}=0.002,{ c}_{0}=0.02,\upmu =0.16,\mathrm{ v}=0.04,\upgamma =0.02,\upvarepsilon =0.7,\mathrm{ d}=0.008,\mathrm{ e}=0.1,\mathrm{ f}=10, { \rho }_{A}=0.03$).

As mentioned in the Methods section, the Turing wavelength was calculated by dividing 2$\uppi$ by the critical wavenumber at which the maximum value of $\mathrm{Re}(\lambda )$ occurred. The function of eigenvalue $\lambda$ on wavenumber $k$ was obtained through dispersion relation analysis. The dispersion relation analysis depends on the A/H submodel (Eqs. 1 and 2) (details are provided in Supplementary Information). The A/H model includes parameters ${\rho }_{A}\mathrm{ and }{\rho }_{H}$ directly. Therefore, the functional relationship between the Turing wavelength and parameters ${\rho }_{A}\mathrm{ and }{\rho }_{H}$ can be obtained by dispersion relation analysis. The function curves of the Turing wavelength on the parameters ${\rho }_{A}\mathrm{ and }{\rho }_{H}$ are shown in Fig. 5a,b, respectively.

The function curve in Fig. 5a shows that parameter ${\rho }_{A}$ has a nonmonotonic effect on the Turing wavelength. With increasing ${\rho }_{A}$, the Turing wavelength decreases first and then increases. In Fig. 5b, the function curve shows that parameter ${\rho }_{H}$ has a monotonic effect on the Turing wavelength. The Turing wavelength increases with increasing ${\rho }_{H}$.

Parameter $\upvarepsilon$ was not able to obtain the functional relationship between the Turing wavelength and $\upvarepsilon$ directly because it was not included in the A/H submodel. However, as mentioned in the Methods section, the A/H submodel was decoupled from the whole model by making *S* and *Y* controllable parameters in the A/H subsystem. When calculating the Turing wavelength through dispersion relation analysis, the selected (S, Y) pair satisfying Turing instability should be provided to the A/H subsystem. It is worth noting that the SY curve of the cell differentiation trajectory was provided by the S/Y subsystem and closely related to parameters $\upvarepsilon$. Therefore, our strategy was to obtain the functional relations between the (S, Y) pair and parameter $\upvarepsilon$ first and then substitute them into the dispersion relation analysis to obtain the relationship between the Turing wavelength and $\upvarepsilon$.

**Figure 6:**
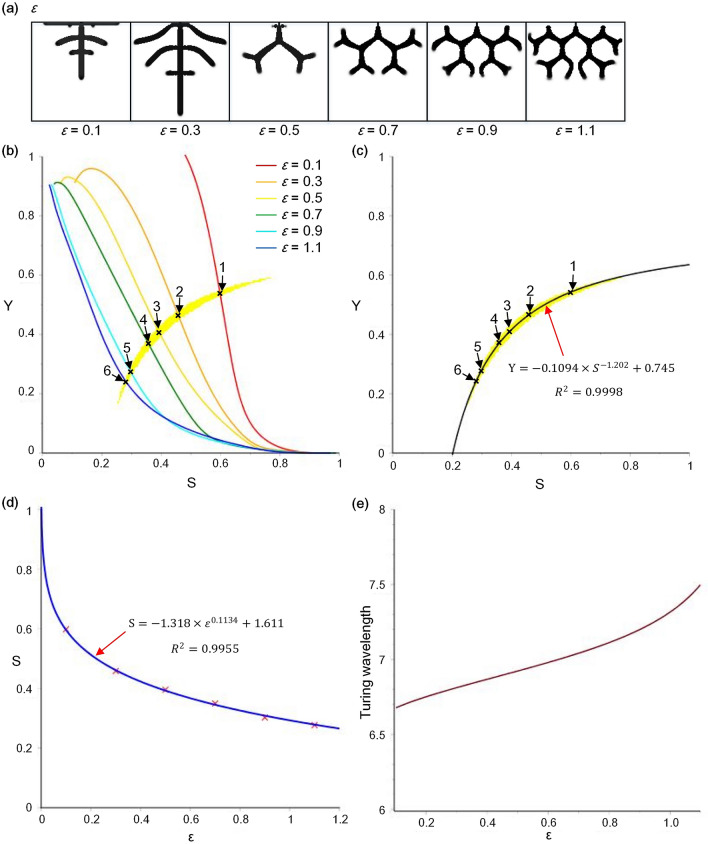
Relationship between the Turing wavelength and parameter $\upvarepsilon$ based on curve fitting and dispersion relation analysis. (**a**) The simulation results of structure formation under parameter $\upvarepsilon$ ranging from 0.1 to 1.1 at intervals of 0.2. (Other parameters:${D}_{A}=0.02, { D}_{H}=0.26, { D}_{s}=0.06,\mathrm{ c}=0.002,{ c}_{0}=0.02,\upmu =0.16,\mathrm{ v}=0.04,\upgamma =0.02,\mathrm{ d}=0.008,\mathrm{ e}=0.1,\mathrm{ f}=10, { \rho }_{A}=0.03, {\rho }_{H}=0.00008$) (**b**) The (S, Y) pairs selected at the centre of overlapping segments of SY-curves and Turing instability region, which correspond to different values of parameter $\upvarepsilon$. The (S, Y) pairs are marked by symbol ‘x’ (shown as points 1–6). The yellow crescent-shaped region is the Turing instability region. The curves of different colours are SY curves of the cell differentiation trajectory in the structures corresponding to different values of parameter $\upvarepsilon$. (**c**) Curve fitting between *Y* and *S*. (**d**) Curve fitting between *S* and $\upvarepsilon$. (**e**) Function curve of the Turing wavelength on parameter $\upvarepsilon$ through dispersion relation analysis.

In this section, the structures were performed with parameter $\upvarepsilon$ ranging from 0.1 to 1.1 at intervals of 0.2 in a square domain, as shown in Fig. 6a. With increasing $\upvarepsilon$, the structure changed from the mode of side branching to tip bifurcation. The crescent-shaped Turing instability region of the model and the SY curves of the cell differentiation trajectory in these structures are shown in Fig. 6b. The (S, Y) pairs (marked by the symbol ‘x’ in Fig. 6b) satisfying Turing instability under different values of $\upvarepsilon$ were extracted from the centre of overlapping segments of the SY-curves and Turing instability region. Through curve fitting based on the extracted (S, Y) pairs and the values of corresponding parameter $\upvarepsilon$, the fitting function between *Y* and *S* was $\mathrm{Y}=-0.1094\times {S}^{-1.202}+0.745$, the goodness of fit was ${R}^{2}=0.9998$, the fitting function between *S* and $\upvarepsilon$ was $\mathrm{S}=-1.318\times {\varepsilon }^{0.1134}+1.611$, and the goodness of fit was ${R}^{2}=0.9955$. The curves of the Y–S fitting function and S–$\upvarepsilon$ fitting function are shown in Fig. 6c,d, respectively. Then, parameter $\upvarepsilon$ could be included in the A/H submodel by substituting the fitting functions. The functional relationship between the Turing wavelength and parameter $\upvarepsilon$ could be obtained by dispersion relation analysis. The function curve is shown in Fig. 6e, revealing a monotonically increasing curve, which means that the Turing wavelength increased with an increasing parameter $\upvarepsilon$.

### The Turing wavelength regulates meshwork pattern formation

According to the functional relationships between the Turing wavelength and model parameters (Figs. 5, 6e), the Turing wavelength could be regulated by the model parameters. The influences of the Turing wavelength on meshwork pattern formation are explored in this section. A square domain with two vertical initial growth points is set as the simulation condition for the local meshwork pattern formation.

**Figure 7:**
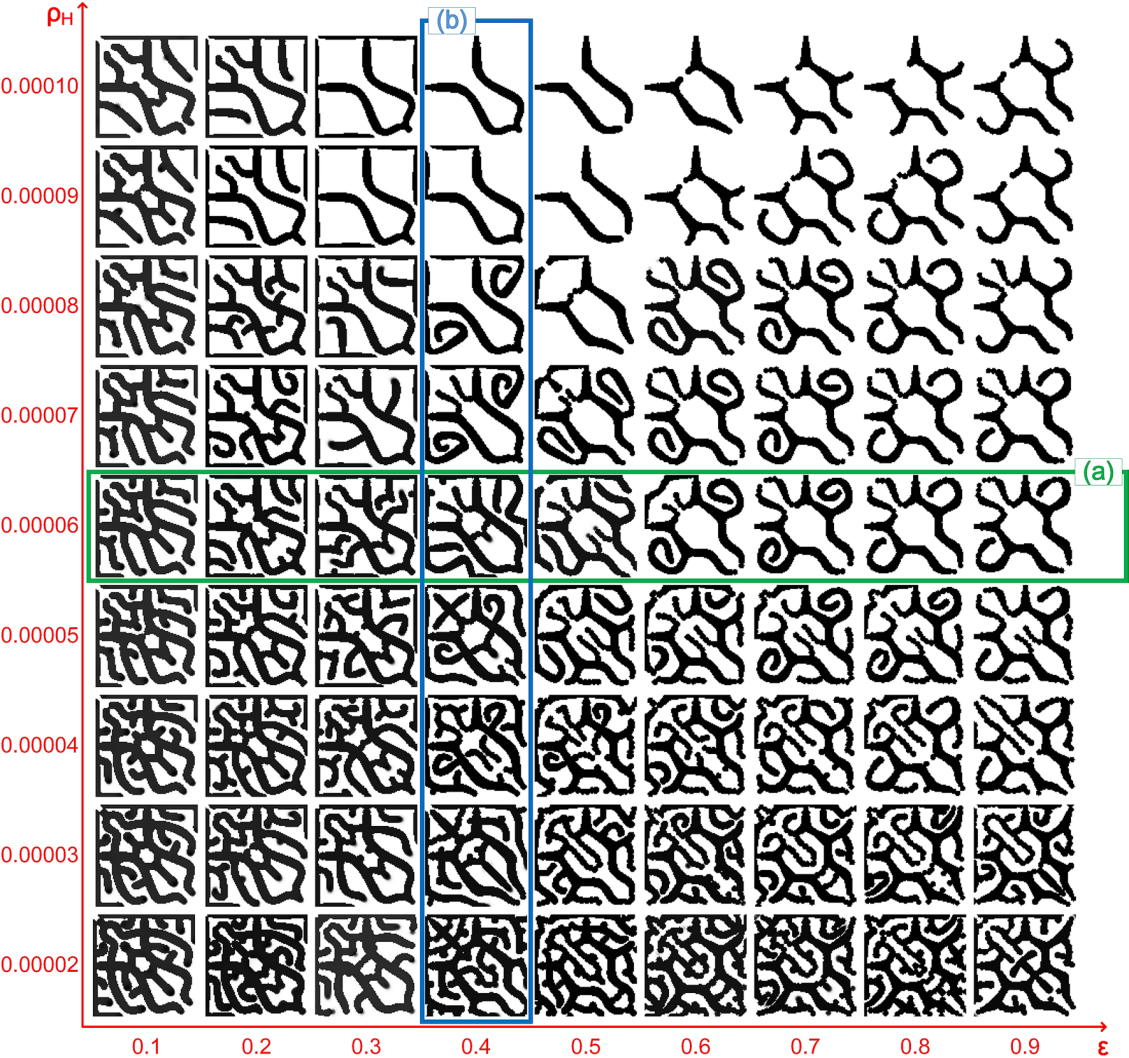
Simulation results of local meshwork pattern formation under variable parameters $\upvarepsilon$ and ${\rho }_{H}$. $\upvarepsilon$ ranges from 0.1 to 0.9 at intervals of 0.1, and ${\rho }_{H}$ ranges from 0.00002 to 0.00010 at intervals of 0.00001. (**a**) Example of the influence of parameter $\upvarepsilon$ on meshwork patterns when ${\rho }_{H}=0.00006$. (**b**) Example of the influence of parameter $\upvarepsilon$ on meshwork patterns when $\upvarepsilon =0.4$. Grid size is 100 × 100. (Other parameters:${D}_{A}=0.02, { D}_{H}=0.26, { D}_{s}=0.06,\mathrm{ c}=0.002,=0.02,\upmu =0.16,\mathrm{ v}=0.04,\upgamma =0.02,\mathrm{ d}=0.008,\mathrm{ e}=0.1,\mathrm{ f}=10, { \rho }_{A}=0.03$).

The simulation results of structure formation with different values of parameters $\upvarepsilon$ and ${\rho }_{H}$ are shown in Fig. 7, as parameters $\upvarepsilon$ and ${\rho }_{H}$ have monotonous effects on the Turing wavelength. Thus, when parameters $\upvarepsilon$ and ${\rho }_{H}$ were small, the number of branches in the structure was large, branch tip fusion occurred frequently, and the level of the meshwork structure was high, while when parameters $\upvarepsilon$ and ${\rho }_{H}$ were large, the branches in the structure were sparse, almost no branch tip fusion behaviour occurred, and the level of the meshwork structure was low. According to the functional relationships between the model parameters and Turing wavelength, the Turing wavelength was small when parameters $\upvarepsilon$ and ${\rho }_{H}$ were small, while the Turing wavelength was larger when parameters $\upvarepsilon$ and ${\rho }_{H}$ were larger. Therefore, we concluded that a small Turing wavelength facilitated dense meshwork pattern formation. Moreover, according to the correspondence between stalk behaviours and Turing wavelength, branch tip fusion corresponded to a small wavelength. As branch tip fusion facilitates meshwork pattern formation^1^, it can be inferred that branch tip fusion occurred frequently for dense meshwork structure formation with a small Turing wavelength. Surprisingly, the simulation results in Fig. 7 were completely consistent with the prediction.

In addition, more details in Fig. 7 confirmed the correspondence between stalk behaviours and Turing wavelength and the functional relationship between model parameters ($\upvarepsilon$ and ${\rho }_{H}$) and the Turing wavelength. For example, Fig. 7a shows the meshwork patterns affected by parameter $\upvarepsilon$. When $\upvarepsilon$ was small, a large number of side branches were generated, and branch tip fusion occurred frequently, leading to dense meshwork structure formation. With the increase in $\upvarepsilon$, the behaviour of side branching gradually changed into tip bifurcation, the number of branch tip fusions was reduced, and the meshwork structure became sparse. According to the relationships between parameter $\upvarepsilon$, Turing wavelength and stalk behaviours, the Turing wavelength increased with the increase in parameter $\upvarepsilon$, and the Turing wavelength corresponding to the stalk behaviour of tip fusion, side branching and tip bifurcation increased in sequence. Therefore, the phenomena of side branching turning into tip bifurcation and reduction of branch tip fusion behaviour with increasing $\upvarepsilon$ in Fig. 7a were consistent with the relationships between parameter $\upvarepsilon$, Turing wavelength and stalk behaviours. For another example, Fig. 7b shows the meshwork patterns affected by parameter ${\rho }_{H}$. With the increase in ${\rho }_{H}$, the number of branches in the structure decreased, the branch tip fusion behaviour decreased, and the meshwork structure became sparse. This phenomenon was also consistent with the relationship between parameter ${\rho }_{H}$, Turing wavelength and stalk behaviours: the larger ${\rho }_{H}$ was, the larger was the Turing wavelength, and the behaviour of branch tip fusion was reduced with a larger Turing wavelength.

**Figure 8:**
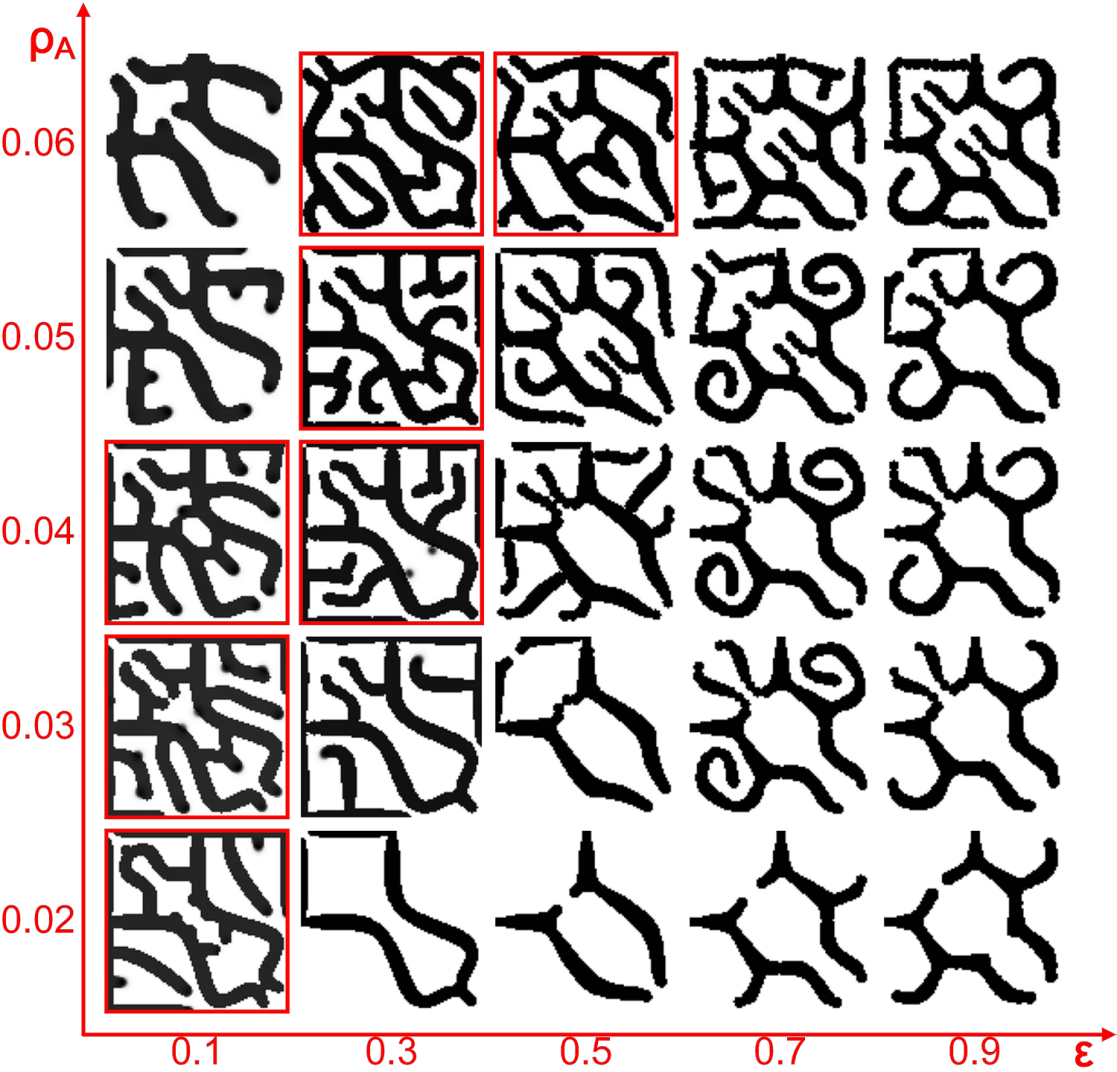
Simulation results of local meshwork pattern formation under variable parameters $\upvarepsilon$ and ${\rho }_{A}$. $\upvarepsilon$ ranges from 0.1 to 0.9 at intervals of 0.2, and ${\rho }_{A}$ ranges from 0.02 to 0.06 at intervals of 0.01. The red square boxes indicate the meshwork structures, including the branch tip fusion behaviour and a large number of branches. Grid size is 100 × 100. (Other parameters:${D}_{A}=0.02, { D}_{H}=0.26, { D}_{s}=0.06,\mathrm{ c}=0.002,=0.02,\upmu =0.16,\mathrm{ v}=0.04,\upgamma =0.02,\mathrm{ d}=0.008,\mathrm{ e}=0.1,\mathrm{ f}=10, {\rho }_{H}=0.00008$).

As parameter ${\rho }_{A}$ had nonmonotonic effects on the Turing wavelength, it might have irregular influences on meshwork pattern formation. The simulation results of structure formation with different values of parameters $\upvarepsilon$ and ${\rho }_{A}$ are shown in Fig. 8. Among the simulation results, the meshwork structures with better quality are marked by red square boxes, in which branch tip fusion occurred and there were many branches. The distribution of these meshwork structures was irregular. For example, a small ${\rho }_{A}$ corresponded to good meshwork structures when $\upvarepsilon =0.1$, while a larger ${\rho }_{A}$ corresponded to good meshwork structures when $\upvarepsilon =0.3\mathrm{ or }0.5$. This phenomenon was probably related to the nonmonotonic functional relationship between ${\rho }_{A}$ and the Turing wavelength, indicating that parameter ${\rho }_{A}$ was not good for controllable regulation of meshwork patterns.

## Discussion

The meshwork pattern is a very important pattern for the development of biological organs and tissues, such as the alveolar microvascular network, which is essential for nutrient transport and gas exchange. This paper aims to explore the Turing mechanism underlying meshwork pattern formation. During the dynamic process of meshwork pattern formation, we found that the meshwork pattern is formed by four kinds of stalk behaviours: stalk extension, tip bifurcation, side branching and tip fusion. We determined the Turing-type patterns corresponding to the four stalk behaviours and found that they were all Turing spot patterns, which indicated that the Turing-type pattern underlying the meshwork pattern was a Turing spot pattern and that the activator peaks produced by Turing instability guided the formation of meshwork patterns. Then, we obtained the Turing wavelength corresponding to each stalk behaviour through statistical evaluation. We found that the Turing wavelength decreased in turn from tip bifurcation to side branching to tip fusion. Through dispersion relation analysis, we also obtained the functional relationships between the Turing wavelength and model parameters ($\upvarepsilon ,{ \rho }_{A}$ and ${\rho }_{H}$). We found that parameters $\upvarepsilon$ and ${\rho }_{H}$ had monotonic effects on the Turing wavelength and that parameter ${\rho }_{A}$ had nonmonotonic effects. Then, we regulated the meshwork patterns by the Turing wavelength by controlling the model parameters. We performed simulations of the local meshwork pattern formation under variable values of model parameters. The simulation results were consistent not only with the relationship between the Turing wavelength and model parameters, but also with the correspondence between the Turing wavelength and stalk behaviours. We concluded that the meshwork pattern was regulated by the Turing wavelength and that a small Turing wavelength facilitated dense meshwork pattern formation.

In this paper, we found that the Turing-type pattern underlying the meshwork pattern was a Turing spot pattern. The spot pattern was in the form of concentration peaks. It induced activator concentration peak formation in the structures. The activator peak activated the irreversible differentiation of cells, and the migration of activator peaks guided the growth of stalks. This result indicated that activator peaks played a key role in the formation of meshwork patterns, which is consistent with a previous study of branching patterns^17^, showing that the activator peak at the branch tip guided the growth of branches. As activator peak formation relies on the Turing spot pattern, these findings indicated that Turing instability of the spot pattern guided the formation of meshwork patterns, which might represent the mathematical mechanism by which genes control the formation of network structures.

Our simulation result of the local meshwork pattern included various stalk behaviours: stalk extension, tip bifurcation, side branching, and branch tip fusion. These stalk behaviours corresponded sequentially to the branching behaviours in biological networks. For example, stalk extension corresponds to sprout growth, such as the sprout growth of a mosaic embryoid body in vitro^18,19^; tip bifurcation corresponds to sprout splitting, such as arterial arborescence in the intracortical capillary networks in the temporal lobe^20^; side branching corresponds to sprouting, such as intersegmental vessel (ISV) sprouting from the dorsal aorta in zebrafish embryos^18,21^; and branch tip fusion corresponds to anastomosis, such as vessel fusion of the dorsal longitudinal anastomotic vessel (DLAV) in zebrafish embryos^21^. The simulation result of the meshwork pattern confirmed that meshwork pattern formation was a complicated process and was formed by the four stalk behaviours.

In this paper, we explored the Turing wavelength corresponding to the four stalk behaviours. The Turing wavelength of each stalk behaviour was obtained by dispersion relation analysis of typical sites in the structures, which is quite different from a previous study on the Turing mechanism of branching patterns^17^. In that study, a whole branching pattern corresponds to a certain Turing wavelength, even for a mixed branching pattern including different branching behaviours (the coexistence of tip bifurcation and side branching). However, this was not sufficiently accurate because the Turing wavelength between stalk behaviours might be obviously different, although the model parameters were the same, such as the different spot densities of Turing patterns shown in Fig. 2d–f. Therefore, typical sites (representing stalk behaviours) were selected for accurate calculation of the Turing wavelengths of stalk behaviours in this paper. These typical sites are on the stalk extension trajectory: the bifurcation site where one stalk splits into two, the sprouting site where a new lateral branch occurs, and the fusion site where two stalks merge into one.

In this paper, we found that parameters $\upvarepsilon$ and ${\rho }_{H}$ had monotonic increasing effects on the Turing wavelength. The meshwork pattern was regulated by controlling parameters $\upvarepsilon$ and ${\rho }_{H}$. However, the detailed effects of parameters $\upvarepsilon$ and ${\rho }_{H}$ on meshwork structures were different. With the increase in ${\rho }_{H}$, the number of branches was reduced, and with the increase in $\upvarepsilon$, the stalk behaviour changed from side branching to tip bifurcation. This means that regulation of the Turing wavelength on the meshwork pattern could be realized in various ways. The different regulatory mechanisms may correspond to different gene functions in organisms. Therefore, it will be important to explore and classify the genes affecting meshwork patterns in the future, which has the potential to thoroughly elucidate the mechanism of meshwork pattern formation.

We also found that parameter ${\rho }_{A}$ had a nonmonotonic function on the Turing wavelength and had irregular influences on meshwork patterns. We obtained the Turing instability regions of the model under different values of parameter ${\rho }_{A}$ (see Supplementary Fig. S6). This result showed that the position and size of the Turing instability region changed greatly with a slight change in parameter ${\rho }_{A}$. We suppose that the high sensitivity of parameter ${\rho }_{A}$ to Turing regions was closely related to its irregular influences on meshwork patterns, meriting for further research in the future.

We propose that the morphogens bone morphogenetic protein-4 (BMP4), matrix carboxyglutamic acid protein (MGP) and fibroblast growth factor 10 (FGF10) corresponded to the activator, inhibitor and substrate of the model, respectively. Since BMP4 has autostimulatory positive feedback^24^, the expression of BMP4 is positively stimulated by FGF10^25,26^, MGP is a well-known inhibitor of BMP4^27,28^, BMP4 induces the expression of MGP^29^, and BMP4 signalling activates vascular cell differentiation and vascular formation^30^. We believe that this set of morphogens will help biologists verify our work in biological experiments.

## Supplementary Information

Supplementary Information.
