# Supplementary material for: Wavelength of a Turing-type mechanism regulates the morphogenesis of meshwork patterns
Source: Sci Rep. 2021 Mar 1;11:4813. doi: 10.1038/s41598-021-84313-7 (PMC7921672; doi:10.1038/s41598-021-84313-7)
Supplement: Supplementary file 1 — Supplementary Information. [file 41598_2021_84313_MOESM1_ESM.pdf]

Wavelength of a Turing-Type Mechanism Regulates the Morphogenesis of Meshwork Patterns

Shan Guo,[1,2] Ming-zhu Sun, [1,2] and Xin Zhao[*1,2]

[1]Institute of Robotics and Automatic Information Systems, Nankai University, Tianjin 300350, China

[2]Tianjin Key Laboratory of Intelligent Robotics, Nankai University, Tianjin 300350, China

*Corresponding author: Zhao Xin, E-mail address: zhaoxin@nankai.edu.cn, Full post address: 201-02, College of Artificial Intelligence, Nankai University, Tianjin 300350, P.R. China

Linear stability analysis of the activator-inhibitor model

To calculate the S-Y parameter space of the activator-inhibitor model for the Turing instability, linear stability analysis [1-5] is performed. The activator-inhibitor model is defined by

$$\begin{cases} \dfrac{\partial A}{\partial t} = \dfrac{cA^2 S}{H} - \mu A + \rho_A Y + D_A \nabla^2 A \\ \dfrac{\partial H}{\partial t} = cA^2 S - vH + \rho_H Y + D_H \nabla^2 H \end{cases} \tag{A1}$$

Denote

$$f(A,H) = \frac{cA^2 S}{H} - \mu A + \rho_A Y, \quad g(A,H) = cA^2 S - vH + \rho_H Y. \tag{A2}$$

We are interested in the positive equilibrium of system (A1). By solving the relation:

$$\begin{cases} f(A,H) = 0 \\ \mathrm{g}(A,H) = 0 \end{cases} \tag{A3}$$

We can obtain the positive equilibrium denoted by $E^* = \begin{pmatrix} A^* \\ H^* \end{pmatrix}$. Then, we make the following nonuniform perturbations from E^*:

$$\begin{pmatrix} A \\ H \end{pmatrix} = \begin{pmatrix} A^* \\ H^* \end{pmatrix} + \zeta \begin{pmatrix} A_k \\ H_k \end{pmatrix} e^{\lambda t + i\boldsymbol{kr}} + c.c. + O(\zeta^2), \tag{A4}$$

where λ is the growth rate of the perturbations over time t, i is an imaginary unit, $\boldsymbol{k}$ is the wave vector, $\boldsymbol{r}$ is the spatial vector in two-dimensional space and c.c. represents the complex conjugate. By substituting Eq. (A4) into system (A1), we obtain the characteristic equation:

$$\begin{pmatrix} r_{11} - k^2 D_A - \lambda & r_{12} \\ r_{21} & r_{22} - k^2 D_H - \lambda \end{pmatrix} = 0, \tag{A5}$$

where

$$r_{11} = \left.\frac{\partial f}{\partial A}\right|_{A^*,H^*} = \frac{2cA^*S}{H^*} - \mu, \quad r_{12} = \left.\frac{\partial f}{\partial H}\right|_{A^*,H^*} = -\frac{cA^{*2}S}{H^{*2}},$$

$$r_{21} = \left.\frac{\partial g}{\partial A}\right|_{A^*,H^*} = 2cA^*S, \quad r_{22} = \left.\frac{\partial g}{\partial H}\right|_{A^*,H^*} = -v.$$

The characteristic equation is equivalent to the following equation:

$$\lambda^2 - tr_k\lambda + \Delta_k = 0, \tag{A6}$$

where

$$\begin{aligned} tr_k &= r_{11} + r_{22} - k^2(D_A + D_H), \\ \Delta_k &= r_{11}r_{22} - r_{12}r_{21} - k^2(r_{11}D_H + r_{22}D_A) + k^4 D_A D_H \end{aligned} \tag{A7}$$

Eigenvalues λ_k are:

$$\lambda_k = \frac{tr_k \pm \sqrt{tr_k^{\ 2} - 4\Delta_k}}{2} \tag{A8}$$

According to Turing theory, Turing instability occurs in system (A1) if $Re(\lambda_{k=0}) < 0$ and $Re(\lambda_{k\neq0}) > 0$ for some k. These conditions require

$$\begin{aligned} & r_{11} + r_{22} < 0, \\ & r_{11}r_{22} - r_{12}r_{21} > 0, \\ & r_{11}D_H + r_{22}D_A > 0, \\ & (r_{11}D_H + r_{22}D_A)^2 - 4D_A D_H(r_{11}r_{22} - r_{12}r_{21}) > 0. \end{aligned} \tag{A9}$$

We take S and Y as the controlled parameters. Therefore, inequalities (A9) define a domain in (S, Y) parameter space, namely, the Turing region. The expression $\lambda = \lambda(k)$ is called a dispersion relation given by (A8). Analysis of the dispersion relation is thus informative because it shows the features of the spatial patterns.

Supplement figures

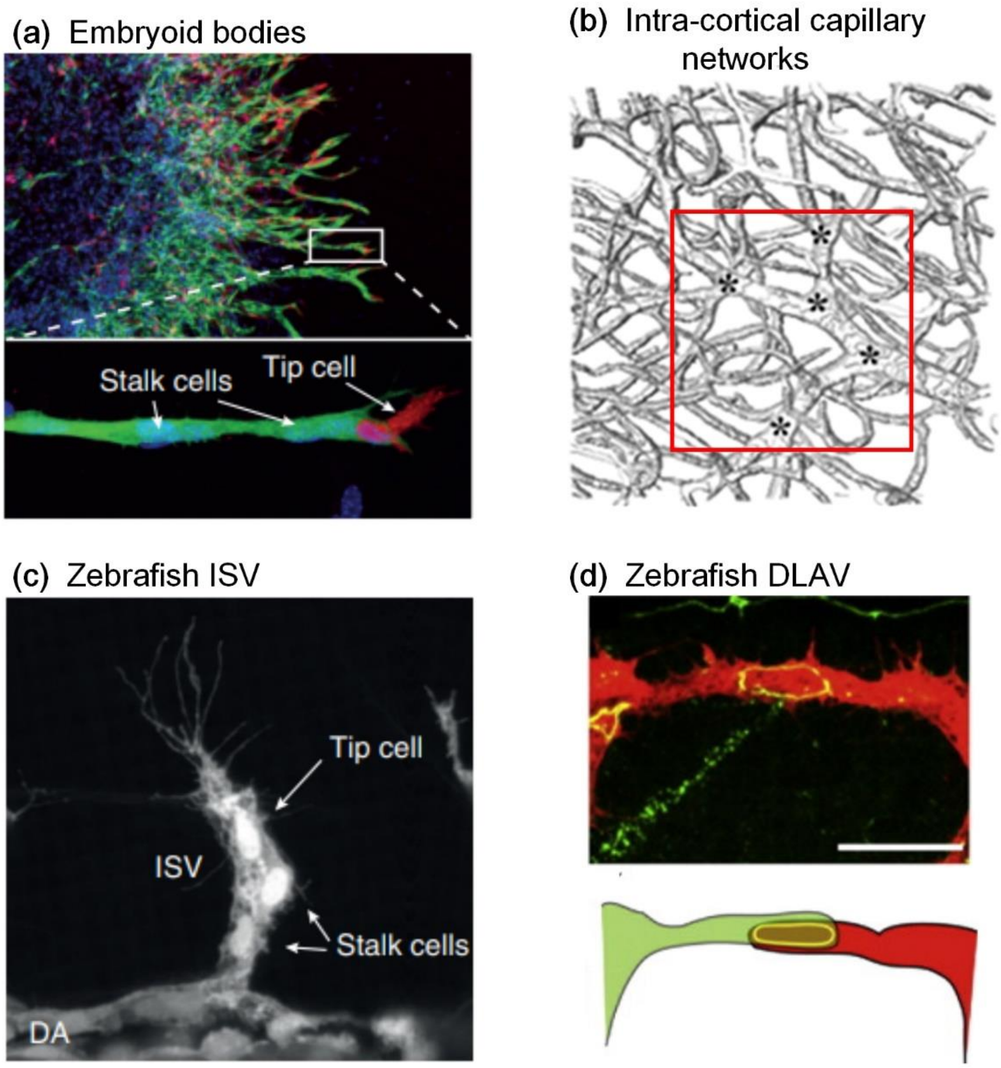

Supplement Figure 1 | Typical stalk behaviors in network structures of biological tissues and organs. (**a**) Sprout growth of a mosaic embryoid body in vitro; (**b**) Arterial arborescence in the intra-cortical capillary networks in a lateral region of the collateral sulcus in the temporal lobe; (**c**) Intersegmental vessel (ISV) sprouting from the dorsal aorta in zebrafish embryo; (**d**) Vessel fusion of the dorsal longitudinal anastomotic vessel (DLAV) in zebrafish embryo.

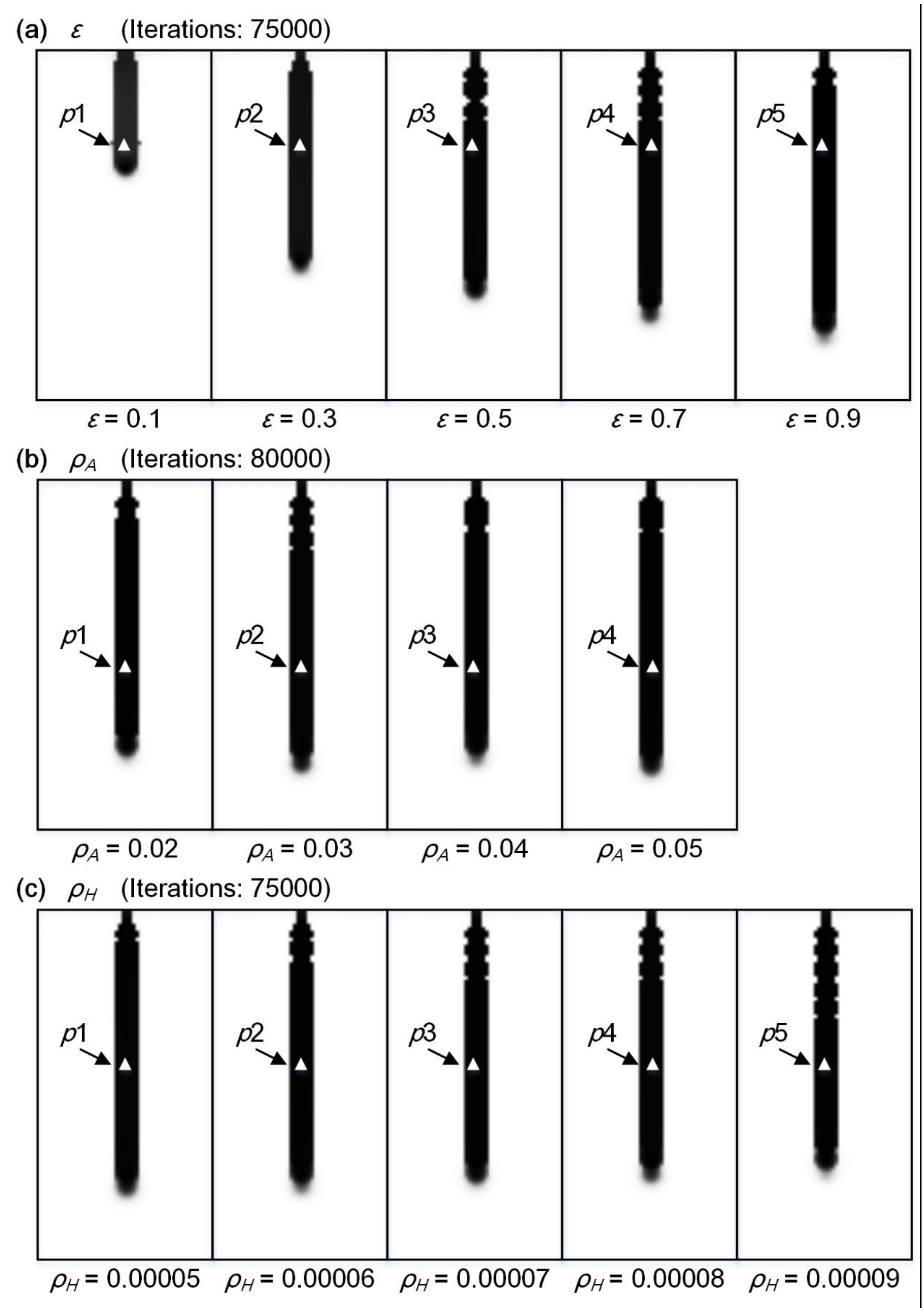

Supplement Figure 2 | Simulation results of stalk extension influenced by parameters ε, ρ_A and ρ_H. (**a**) Results of stalk extension with variable ε after 75000 iterations. Parameter ε changes from 0.1 to 0.9 with 0.2 step size. (Other parameters: $D_A = 0.02$, $D_H = 0.26$, $D_s = 0.06$, $c =$

0.002, $c_0 = 0.02$, $\mu = 0.16$, $v = 0.04$, $\gamma = 0.02$, $d = 0.008$, $e = 0.1$, $f = 10$, $\rho_A = 0.03$, $\rho_H = 0.00008$.) (**b**) Results of stalk extension with variable ρ_A after 80000 iterations. Parameter ρ_A changes from 0.02 to 0.05 with 0.01 step size. (Other parameters: $D_A = 0.02$, $D_H = 0.26$, $D_s = 0.06$, $c = 0.002$, $c_0 = 0.02$, $\mu = 0.16$, $v = 0.04$, $\gamma = 0.02$, $\varepsilon = 0.7$, $d = 0.008$, $e = 0.1$, $f = 10$, $\rho_H = 0.00008$.) (**c**) Results of stalk extension with variable ρ_H after 75000 iterations. Parameter ρ_H changes from 0.00005 to 0.00009 with 0.00001 step size. (Other parameters: $D_A = 0.02$, $D_H = 0.26$, $D_s = 0.06$, $c = 0.002$, $c_0 = 0.02$, $\mu = 0.16$, $v = 0.04$, $\gamma = 0.02$, $\varepsilon = 0.7$, $d = 0.008$, $e = 0.1$, $f = 10$, $\rho_A = 0.03$.) The typical points (marked by white triangles) will be used to calculate Turing wavelength through dispersion relation analysis.

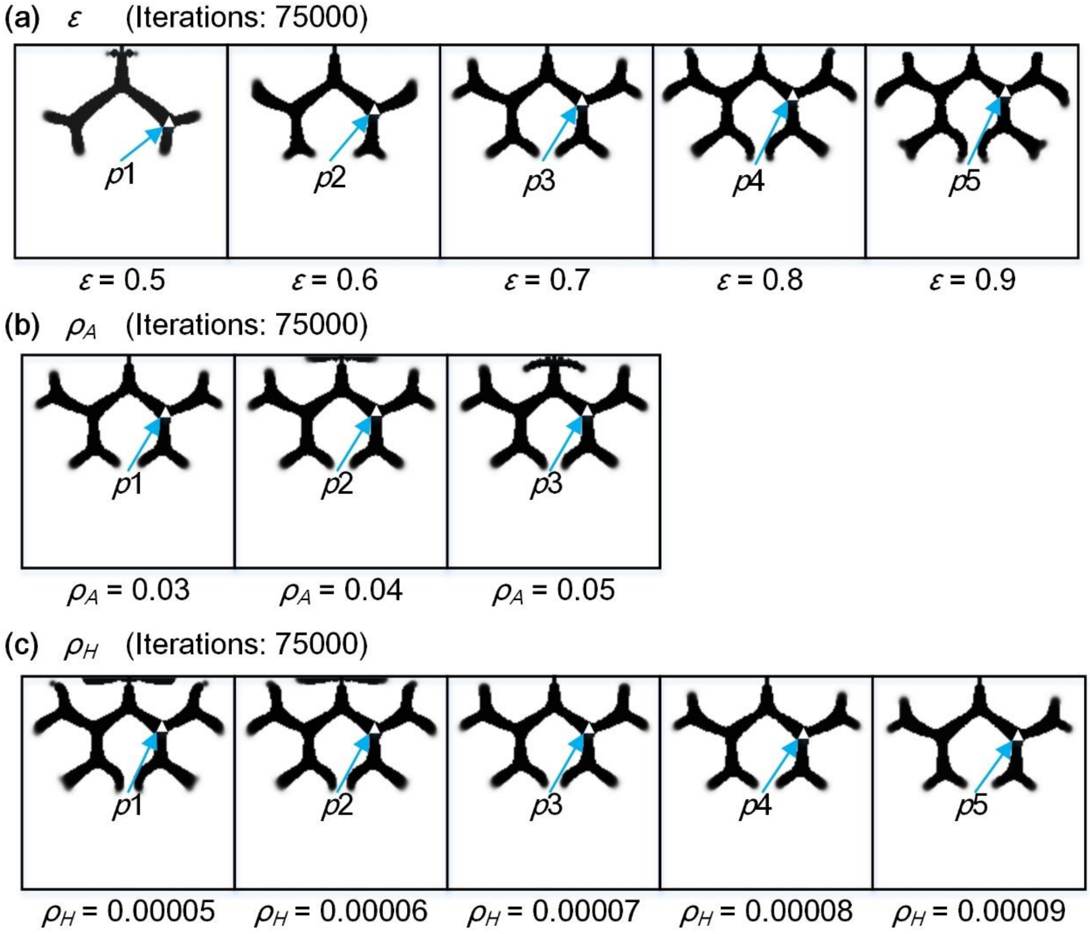

Supplement Figure 3 | Simulation results of tip bifurcation influenced by parameters ε, ρ_A and

$\boldsymbol{\rho_H}$. (**a**) Results of tip bifurcation with variable ε after 75000 iterations. Parameter ε changes from 0.5 to 0.9 with 0.1 step size. (Other parameters: $D_A = 0.02,\ D_H = 0.26,\ D_s = 0.06,\ \mathrm{c} = 0.002,\ c_0 = 0.02,\ \mu = 0.16,\ \mathrm{v} = 0.04,\ \gamma = 0.02,\ \mathrm{d} = 0.008,\ \mathrm{e} = 0.1,\ \mathrm{f} = 10,\ \rho_A = 0.03,\ \rho_H = 0.00008.$) (**b**) Results of tip bifurcation with variable ρ_A after 75000 iterations. Parameter ρ_A changes from 0.03 to 0.05 with 0.01 step size. (Other parameters: $D_A = 0.02,\ D_H = 0.26,\ D_s = 0.06,\ \mathrm{c} = 0.002,\ c_0 = 0.02,\ \mu = 0.16,\ \mathrm{v} = 0.04,\ \gamma = 0.02,\ \varepsilon = 0.7,\ \mathrm{d} = 0.008,\ \mathrm{e} = 0.1,\ \mathrm{f} = 10, \rho_H = 0.00008$.) (**c**) Results of tip bifurcation with variable ρ_H after 75000 iterations. Parameter ρ_H changes from 0.00005 to 0.00009 with 0.00001 step size. (Other parameters: $D_A = 0.02,\ D_H = 0.26,\ D_s = 0.06,\ \mathrm{c} = 0.002,\ c_0 = 0.02,\ \mu = 0.16,\ \mathrm{v} = 0.04,\ \gamma = 0.02,\ \varepsilon = 0.7,\ \mathrm{d} = 0.008,$ $\mathrm{e} = 0.1,\ \mathrm{f} = 10,\ \rho_A = 0.03$.) The typical points (marked by white triangles) will be used to calculate Turing wavelength through dispersion relation analysis.

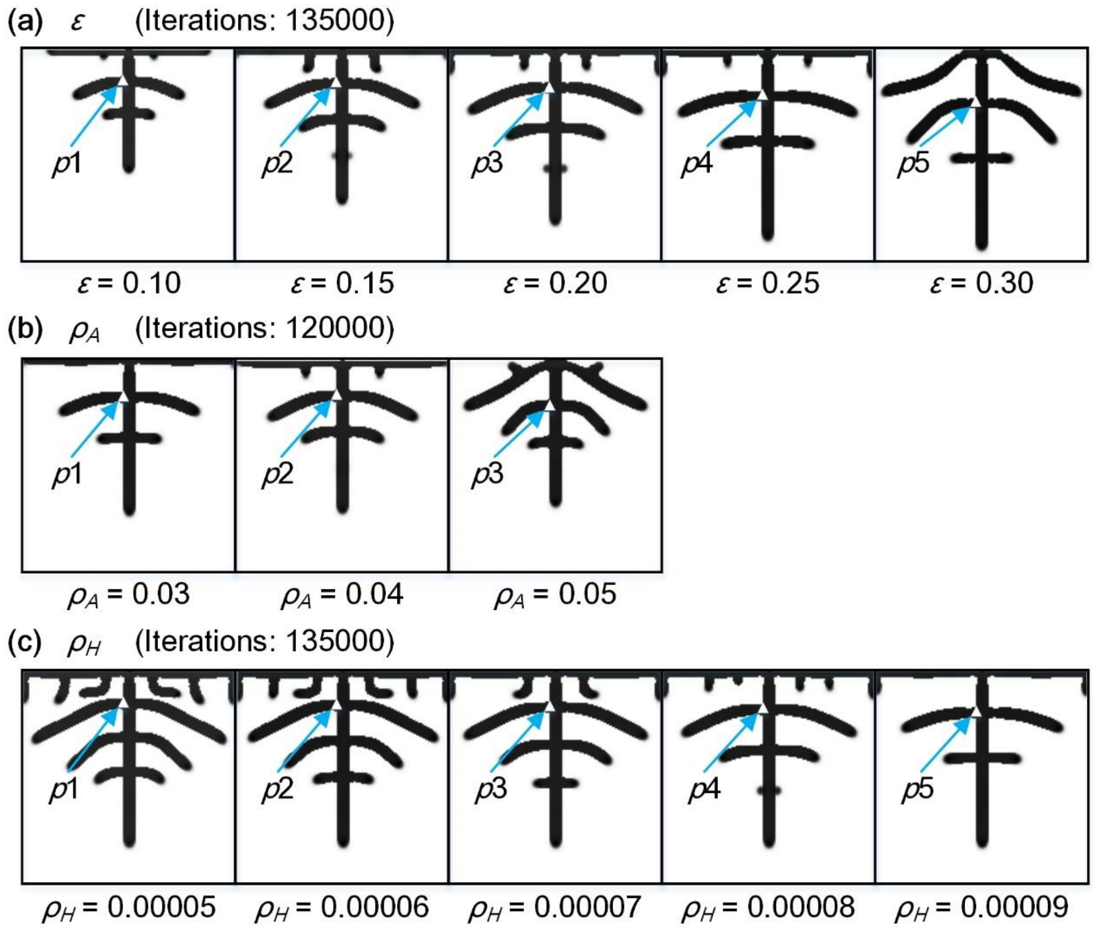

Supplement Figure 4 | Simulation results of side branching influenced by parameters ε, ρ_A and ρ_H. (**a**) Results of side branching with variable ε after 135000 iterations. Parameter ε changes from 0.10 to 0.30 with 0.05 step size. (Other parameters: $D_A = 0.02,\ D_H = 0.26,\ D_s = 0.06,\ \mathrm{c} = 0.002,\ c_0 = 0.02,\ \mu = 0.16,\ \mathrm{v} = 0.04,\ \gamma = 0.02,\ \mathrm{d} = 0.008,\ \mathrm{e} = 0.1,\ \mathrm{f} = 10,\ \rho_A = 0.03,$ $\rho_H = 0.00008$.) (**b**) Results of side branching with variable ρ_A after 120000 iterations. Parameter ρ_A changes from 0.03 to 0.05 with 0.01 step size. (Other parameters: $D_A = 0.02,\ D_H = 0.26,\ D_s = 0.06,\ \mathrm{c} = 0.002,\ c_0 = 0.02,\ \mu = 0.16,\ \mathrm{v} = 0.04,\ \gamma = 0.02,\ \varepsilon = 0.2,\ \mathrm{d} = 0.008,\ \mathrm{e} = 0.1,\ \mathrm{f} = 10, \rho_H = 0.00008$.) (**c**) Results of side branching with variable ρ_H after 135000 iterations. Parameter ρ_H changes from 0.00005 to 0.00009 with 0.00001 step size. (Other parameters: $D_A = 0.02,\ D_H = 0.26,\ D_s = 0.06,\ \mathrm{c} = 0.002,\ c_0 = 0.02,\ \mu = 0.16,\ \mathrm{v} = 0.04,\ \gamma = 0.02,\ \varepsilon = 0.2,$ $\mathrm{d} = 0.008,\ \mathrm{e} = 0.1,\ \mathrm{f} = 10,\ \rho_A = 0.03$.) The typical points (marked by white triangles) will be

used to calculate Turing wavelength through dispersion relation analysis.

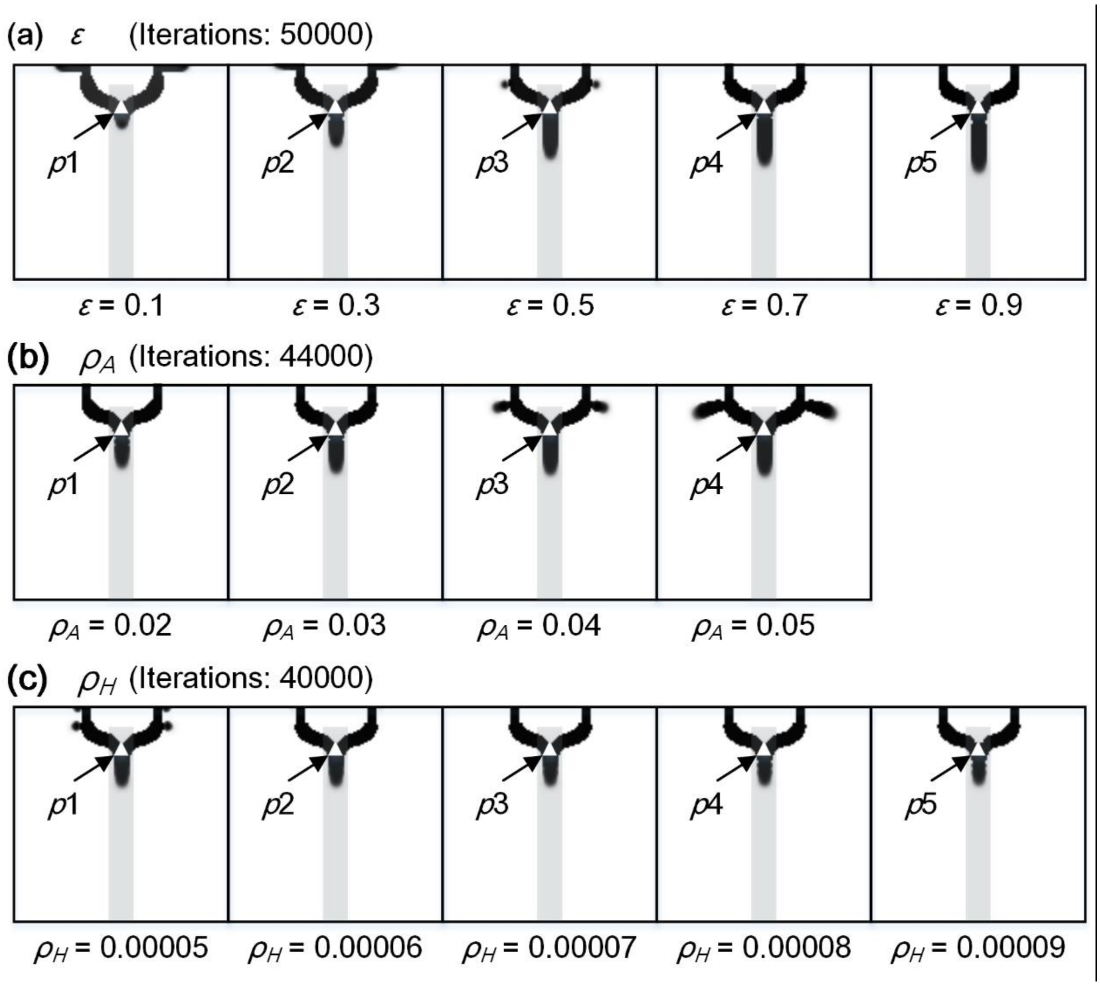

Supplement Figure 5 | Simulation results of tip fusion influenced by parameters ε, ρ_A and ρ_H.

(**a**) Results of side branching with variable ε after 50000 iterations. Parameter ε changes from 0.1 to 0.9 with 0.2 step size. (Other parameters: $D_A = 0.02,\ D_H = 0.26,\ D_s = 0.06,\ \mathrm{c} = 0.002,\ \mu = 0.16,\ \mathrm{v} = 0.04,\ \gamma = 0.02,\ \mathrm{d} = 0.008,\ \mathrm{e} = 0.1,\ \mathrm{f} = 10,\ \rho_A = 0.03,\ \rho_H = 0.00008$.) (**b**) Results of tip fusion with variable ρ_A after 44000 iterations. Parameter ρ_A changes from 0.02 to 0.05 with 0.01 step size. (Other parameters: $D_A = 0.02,\ D_H = 0.26,\ D_s = 0.06,\ \mathrm{c} = 0.002,\ \mu = 0.16,\ \mathrm{v} = 0.04,\ \gamma = 0.02,\ \varepsilon = 0.7,\ \mathrm{d} = 0.008,\ \mathrm{e} = 0.1,\ \mathrm{f} = 10, \rho_H = 0.00008$.) (**c**) Results of tip fusion with variable ρ_H after 40000 iterations. Parameter ρ_H changes from 0.00005 to 0.00009 with 0.00001

step size. (Other parameters: $D_A = 0.02,\ D_H = 0.26,\ D_s = 0.06,\ \mathrm{c} = 0.002,\ \mu = 0.16,\ \mathrm{v} = 0.04,\ \gamma = 0.02,\ \varepsilon = 0.7,\ \mathrm{d} = 0.008,\ \mathrm{e} = 0.1,\ \mathrm{f} = 10,\ \rho_A = 0.03$.) The substrate production rate is set $c_0 = 0.05$ in the high concentration substrate region and $c_0 = 0.02$ in the other region. The typical points (marked by white triangles) will be used to calculate Turing wavelength through dispersion relation analysis.

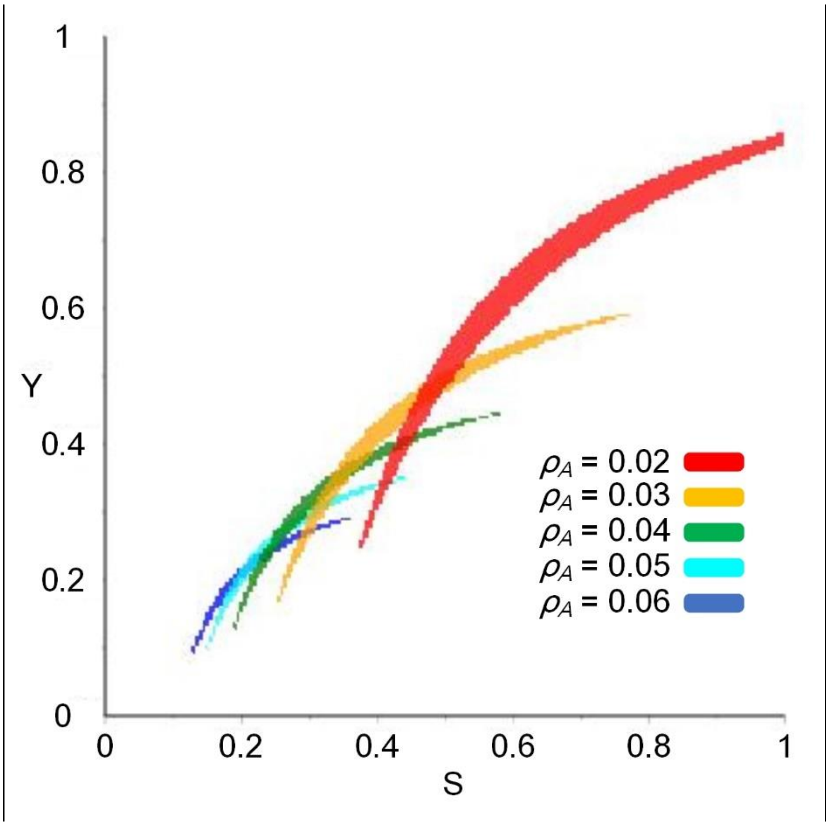

Supplement Figure 6 | Turing instability regions of mathematical model affected by parameter $\boldsymbol{\rho_A}$. The parameter ρ_A ranges from 0.02 to 0.06 at intervals 0.01. (Other parameters: $D_A = 0.02,\ D_H = 0.26,\ D_s = 0.06,\ \mathrm{c} = 0.002,\ c_0 = 0.02,\ \mu = 0.16,\ \mathrm{v} = 0.04,\ \gamma = 0.02,\ \varepsilon = 0.7,\ \mathrm{d} = 0.008,\ \mathrm{e} = 0.1,\ \mathrm{f} = 10, \rho_H = 0.00008$.)

References

[1] Sun GQ, Wang CH, Wu ZY. Pattern dynamics of a Gierer–Meinhardt model with spatial effects. Nonlinear Dynamics. 2017:1–12.

[2] Sun GQ, Jusup M, Jin Z, Wang Y, Wang Z. Pattern transitions in spatial epidemics: Mechanisms and emergent properties. Physics of Life Reviews. 2016.

[3] Sun GQ. Pattern formation of an epidemic model with diffusion. Nonlinear Dynamics. 2012; 69(3):1097–104.

[4] Sun GQ. Mathematical modeling of population dynamics with Allee effect. Nonlinear Dynamics. 2016:1–12.

[5] Sun GQ, Wu ZY, Wang Z, Jin Z. Influence of isolation degree of spatial patterns on persistence of populations. Nonlinear Dynamics. 2016; 83(1–2):811–9.
